# COVID-19 Pandemic: Different Associative Relationships of City Lockdown With Preterm Births in Three Cities – An Ecological Study

**DOI:** 10.3389/fped.2021.644771

**Published:** 2021-04-15

**Authors:** Po-Yin Cheung, Belal Alshaikh, Chuanzhong Yang

**Affiliations:** ^1^Department of Pediatrics, University of Alberta, Edmonton, AB, Canada; ^2^Department of Pediatrics, Cumming School of Medicine, University of Calgary, Alberta, AB, Canada; ^3^Shenzhen Maternity and Child Healthcare Hospital, Southern Medical University, Shenzhen, China

**Keywords:** prematurity, city lockdown, environmental pollution, pandemic (COVID-19), stillbirths

## Abstract

In 2020, the global spread of Severe Acute Respiratory Syndrome Coronavirus 2 (SARS-CoV-2) infection (also known as COVID-19) has led to pandemic health issues with significant changes in individual and community practices. Preterm birth could be one of the risks in pregnant mothers who are infected by the SARS-CoV-2. Preterm births contribute upto 10% of all births and incur significant impact on the child health and cost to the health care system. However, the association of city lockdown during COVID-19 pandemic with the rate of preterm births is unclear. In a cohort study, we examined the association of city lockdown during the COVID-19 pandemic with the births at different gestations in three different cities. Compared with the pre-pandemic epoch, the associative relationships ranged from a decrease in all births, all births across all preterm gestations and to preterm births in moderately and late preterm gestations. We concluded that there were variable associative relationships of city lockdown during COVID-19 pandemic with preterm births. This could be related to the differences in health, societal and cultural factors, which will inspire further studies in this area.

## Introduction

In 2020, the global spread of Severe Acute Respiratory Syndrome Coronavirus 2 (SARS-CoV-2) infection (also known as COVID-19) has led to pandemic health issues with significant changes in individual and community practices. Masking, hygienic practices and city lockdown are advocated to different extents in various jurisdictions. Preterm birth could be one of the risks in pregnant mothers who are infected by the SARS-CoV-2 (1). However, the association of city lockdown during COVID-19 pandemic on the rate of preterm births is controversial.

Handley et al. recently reported the birth outcomes before and during pandemic period of the SARS-CoV-2 infection in two Philadelphia Hospitals (2). They did not detect significant changes in preterm or stillbirth rates during the COVID-19 pandemic in a racially diverse urban cohort. Of note, it was uncertain if there were significant reductions in the community activities in the metropolitan city. Hedermann et al. observed that in a Danish cohort, the rate of extremely premature birth decreased during the COVID-19 lockdown, while there was no significant difference between the lockdown and previous years for other GA categories (3). Recently Khalil et al. published a report and showed an increase in stillbirths during the 4-months pandemic epoch, there was no change in the preterm births and neonatal unit admissions (4). Interestingly, hospitals in some countries observed dramatic decreases in preterm births during the lockdowns (5). In a cohort study before and during the COVID-19 pandemic, we aimed to examine the associative relationship between city lockdown and the rates of preterm births in three different cities, two in Canada (Edmonton and Calgary in the province of Alberta) and one advanced metro city in the People's Republic of China (Shenzhen). We speculated different phenomenon would be observed in these cities.

## Methods

Following institutional approval for a COVID-19 audit project, we accessed the database collected by the provincial and municipal epidemiological programs of Alberta Perinatal Health Program and Shenzhen Pregnancy Outcome Program. We studied the rates of preterm births (<37^+0^ weeks gestation) categorized into different groups according to the range of preterm gestation (<37^+0^ weeks, 32^+0^-36^+6^ weeks, <32^+0^ weeks, 28^+0^-31^+6^ weeks, <28^+0^ weeks) of Edmonton and Calgary in Alberta, Canada and Shenzhen in China. We also examined the rates of stillbirths during these epochs. We further compared the rates of preterm births at different gestational groups and stillbirths (defined as births at ≥20 weeks gestation and/or ≥500g) during pre-pandemic and pandemic epochs using the z-test (SigmaPlot v14, Systat Software Inc., San Jose, CA). Odds ratios and 95% confidence interval were also calculated.

In the province of Alberta, Canada, Edmonton and Calgary have a combined total population of 2.6 million with approximately 35,000 births per year. City lockdown in Alberta was declared on March 12, 2020. In Calgary and Edmonton, Alberta, Canada, pre-pandemic and pandemic epochs were March 1 to April 30, 2019 and March 1 to April 30, 2020, respectively. In Shenzhen, People's Republic of China, pre-pandemic and pandemic epochs were February 1 to February 29, 2019 and February 1 to February 29, 2020, respectively as the city lockdown in Shenzhen was declared on January 23, 2020. Of note, Shenzhen has a resident population of 12.5 million with a significant migrant population. The latter group might have moved out of the city for various reasons including the concurrent Chinese New Year Festival around the time of city-wide lockdown. The association of city lockdown with preterm births on the migratory movements of population during pandemic is uncertain.

## Results

We found that the pattern of reduction in preterm births was different between Edmonton and Calgary in Alberta, although both showed a significant reduction in the number of preterm births, when compared with the corresponding pre-pandemic epochs in 2019 (Table 1). Indeed, there was significant reduction of very preterm and extremely preterm births in Calgary but not in Edmonton where the significant reduction was in births at 32^+0^ to <37^+0^ gestation.

**Table 1:** Birth rates of different gestations and odds ratio [95% confidence interval (CI)] during pre-pandemic and pandemic COVID-19 epochs in Calgary, Edmonton and Shenzhen.

		**Gestational age of preterm births**				
	**Total births**	** <37^**+0**^ weeks (preterm)**	**32^**+0**^-36^**+6**^ weeks**	** <32^**+0**^ weeks (very preterm)**	**28^**+0**^-31^**+6**^ weeks**	** <28^**+0**^ weeks (extremely preterm)**
**Calgary**						
Pre-pandemic	2969	318 (10.7%)	245 (8.3%)	73 (2.5%)	42 (1.4%)	31 (1.0%)
Pandemic	2628	175 (6.7%)*	157 (6.0%)*	18 (0.7%)*	10 (0.4%)*	8 (0.3%)*
Odds ratio [95% CI]		0.59 [0.49, 0.72]	0.71 [0.57, 0.87]	0.27 [0.16, 0.46]	0.27 [0.13, 0.53]	0.29 [0.13, 0.63]
**Edmonton**						
Pre-pandemic	3085	398 (12.9%)	305 (9.9%)	93 (3.0%)	35 (1.1%)	58 (1.9%)
Pandemic	2970	305 (10.3%)*	231 (7.8%)*	74 (2.5%)	33 (1.1%)	41 (1.4%)
Odds ratio [95% CI]		0.77 [0.66, 0.91]	0.77 [0.64, 0.92]	0.82 [0.60, 1.12]	0.98 [0.61, 1.58]	0.73 [0.49, 1.09]
**Shenzhen**						
Pre-pandemic	13776	893 (6.5%)	807 (5.9%)	86 (0.6%)	60 (0.4%)	26 (0.2%)
Pandemic	11727	770 (6.6%)	713 (6.1%)	57 (0.5%)	46 (0.4%)	11 (0.1%)
Odds ratio [95% CI]		1.01 [0.92, 1.12]	1.04 [0.94, 1.15]	0.78 [0.56, 1.09]	0.90 [0.61, 1.32]	0.50 [0.25, 1.01]

* *p < 0.01 (vs. corresponding pre-pandemic epoch, z test). In Calgary and Edmonton, Alberta, Canada, pre-pandemic and pandemic epochs were March 1 to April 30, 2019 and March 1 to April 30, 2020, respectively. City lockdown in Alberta was declared on March 12, 2020. In Shenzhen, People's Republic of China, pre-pandemic and pandemic epochs were February 1 to February 29, 2019 and February 1 to February 29, 2020, respectively. City lockdown in Shenzhen was declared on January 23, 2020*.

Interestingly, in the city of Shenzhen, People's Republic of China, we did not observe any significant reduction in preterm births across all gestations during the pandemic (Table 1), while the total number of births decreased in pandemic epoch.

Regarding the rates of stillbirths in Calgary, Edmonton and Shenzhen, we did not find any significant differences during the pre-pandemic and pandemic epochs (16/2969 and 23/2628; 31/3085 and 29/2970; 21/13776 and 20/11727; *p* = 0.13, 0.91 and 0.72; respectively). The increase in stillbirths in Calgary was modest.

## Discussion

Up to date, there are few reports that investigated the association between adverse birth outcomes and city lockdown during the COVID-19 pandemic period. Matheson et al. recently reported low rates of preterm births during the COVID-19 lockdown in Melbourne, Australia (6). They observed lower rates of prematurity at <28, <34 and <37 weeks gestations with odds ratios of 0.46, 0.71 and 0.81, respectively, in the 2448 births during the pandemic city lockdown period of July-September 2020 when compared with the 2,514 births during July-September 2019 before pandemic. Interestingly, Caniglia et al. observed a modest reduction in adverse birth outcomes (stillbirth, preterm birth, small-for-gestational-age fetuses, and neonatal death) following the COVID-19 lockdown in Botswana (7). This highlights the differential associative relationships in affluent and middle- and low-income countries.

Our findings showed a variable associative relationship of city lockdown with preterm births depending on the societal-cultural and gestational age in different jurisdictions. Hederman et al. suggested that elements of the lockdown including reduced infection load and reduced physical activity might possibly be beneficial for reducing extreme preterm births and potentially reducing infant mortality (3). Interestingly, Aune et al. reported a nonlinear association between physical activity and preterm birth (8). City lockdowns have many potential effects, both stabilizing and triggering, on preterm labor and delivery. These include physical and psychological/mental stressors, domestic violence, and air pollution which all play a role in preterm births (9, 10). However, Wood et al. reported no reduction in the overall rate of preterm birth at <37 weeks of gestation (7.4 and 7.9% of 4,644 and 4,712 births at pre-pandemic and pandemic eras, respectively), nor any differences in the rate of delivery at <34, <32, or <28 weeks in a hospital system in Massachusetts in the United States (11). Although their findings might not support the theory that quarantine or health care delivery changes during the COVID-19 pandemic decreased rates of preterm births, we believe that the discrepant observations illustrate the complex relationships between city lockdowns and preterm births as well as confounding bias and ecological bias in the interpretation of aggregated data. During the COVID-19 pandemic, there are global city lockdowns. Multiple associations between lockdowns and diseases were proposed, and a large-scale multi-centered study will help understanding of the issue, whilst minimizing the publication bias.

In the last year, many reports and systematic reviews have been published to examine the association between COVID-19 in pregnant women and the maternal and neonatal outcomes. Papapanou et al. analyzed 39 reviews and, after accounting for quality of studies, reported increased rates of cesarean sections and preterm birth rates, with iatrogenic reasons potentially involved (12). Further, in cases of symptomatic women with confirmed infection, there are concerns regarding high maternal and neonatal ICU admission. The probability of vertical transmission cannot be excluded. Based on population-based cohort studies and systematic reviews, COVID-19 during pregnancy is associated with increased risks of adverse birth outcomes including preterm births and cesarean section deliveries without significant increase in the risk for low birth weight, asphyxia and other neonatal morbidity (13–15). Interestingly, Al-Lami et al. suggested the biological evidence that SARS-CoV-2 might cause preterm labor in pregnant women with no medical indication for preterm labor induction and be related to the hypertensive disorders of pregnancy (16). While there is little evidence on the maternal-fetal vertical transmission of SARS-CoV-2, the neonatal SARS-CoV-2 infection is usually mild (15, 17–19). Controversies exist regarding the vaccination in pregnant and lactating women who are at risk for COVID-19 infection with possible effects on her developing fetus or newborn (20). Furthermore, given the unknown disease course, it is important to monitor the long-term health effects of SARS-CoV-2 infection on pregnant women and their children.

Preterm birth occurs at ~10% of births worldwide and is associated with significant mortality, acute morbidity and long-term neurodevelopmental and neurobehavioral consequences. Razzaque et al. advocated the importance of antenatal care visits for maternal and fetal health with regular consultation with a health professional throughout the pregnancy for mothers with SARS-CoV-2 infection (21). We fully agree in view of recent reports on adverse neonatal outcomes in maternal SARS-CoV-2 infection. On the other hand, significant health care expenses are attributed to the care of preterm infants including further educational and social costs in the subsequent years.

Our study is limited by the retrospective review of epidemiological databases with an one-time snap-shot observation. We have challenges and should be cautious in the interpretation of aggregated data which have significant ecological fallacy and confounding bias of individual data (22). Of note, preterm births are associated with high rates of stillbirths. There is a speculation that mothers are afraid to seek medical attention out of the fear of SARS-CoV-2 infection, which may affect the rate of stillbirths. While we found no significant increase in the rate of stillbirths during the COVID-19 pandemic, cautious interpretation of our findings is required because the data from rural communities that these urban centers serve were not available at the time of publication. Further, the associative relationship for all three cities combined were not calculated due to the heterogeneity of the characteristics of population, culture, social and healthcare systems.

Our interesting findings are preliminary and needed to be confirmed with the data from other jurisdictions. The causes of variations in the rate of preterm births between different regions are yet to be determined. Responses to the lockdown restrictions, air pollution levels, social safety network for pregnant women and government measures to relieve financial stress on families are potential factors. Understanding the relationship between lockdown and preterm births may help develop policies to prevent and reduce preterm births.

## Data Availability Statement

The original contributions generated for the study are included in the article/Supplementary Material, further inquiries can be directed to the corresponding authors.

## Author Contributions

P-YC conceptualized and designed the study, collected and analyzed the data, drafted the initial manuscript, and reviewed and revised the manuscript. BA and CY conceptualized the study, collected the data, and critically reviewed the manuscript for important intellectual content. All authors contributed to the article and approved the submitted version.

## Conflict of Interest

The authors declare that the research was conducted in the absence of any commercial or financial relationships that could be construed as a potential conflict of interest.
