# Supplementary material for: COVID-19 Pandemic: Different Associative Relationships of City Lockdown With Preterm Births in Three Cities – An Ecological Study
Source: Front Pediatr. 2021 Apr 15;9:644771. doi: 10.3389/fped.2021.644771 (PMC8081901; doi:10.3389/fped.2021.644771)
Supplement: Supplementary file 1 [file Data_Sheet_1.pdf]

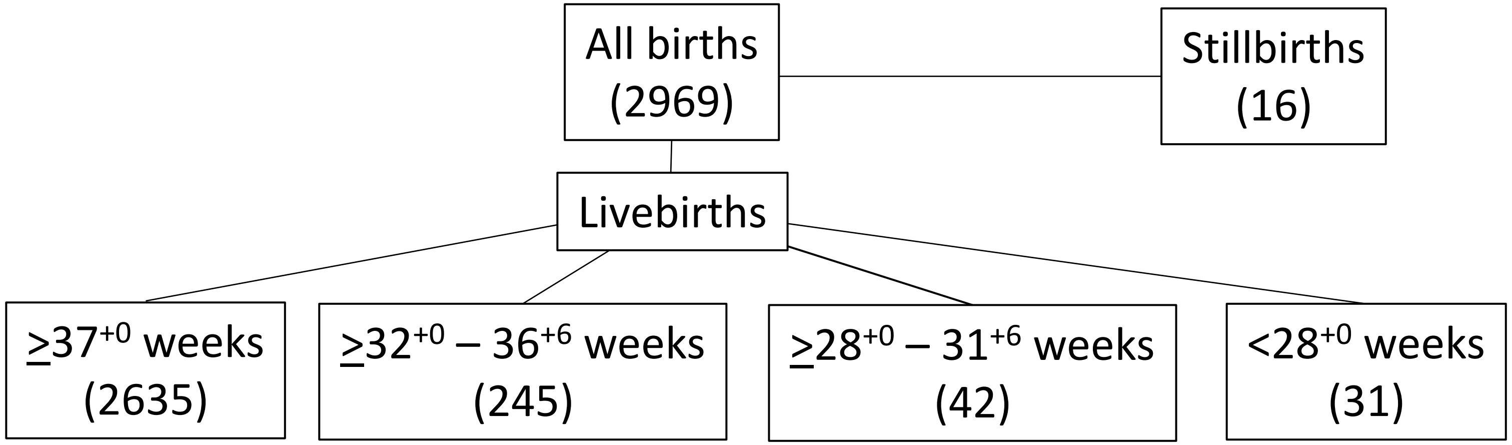

Data of Calgary during pre-pandemic epoch [March 1 to April 30, 2019]

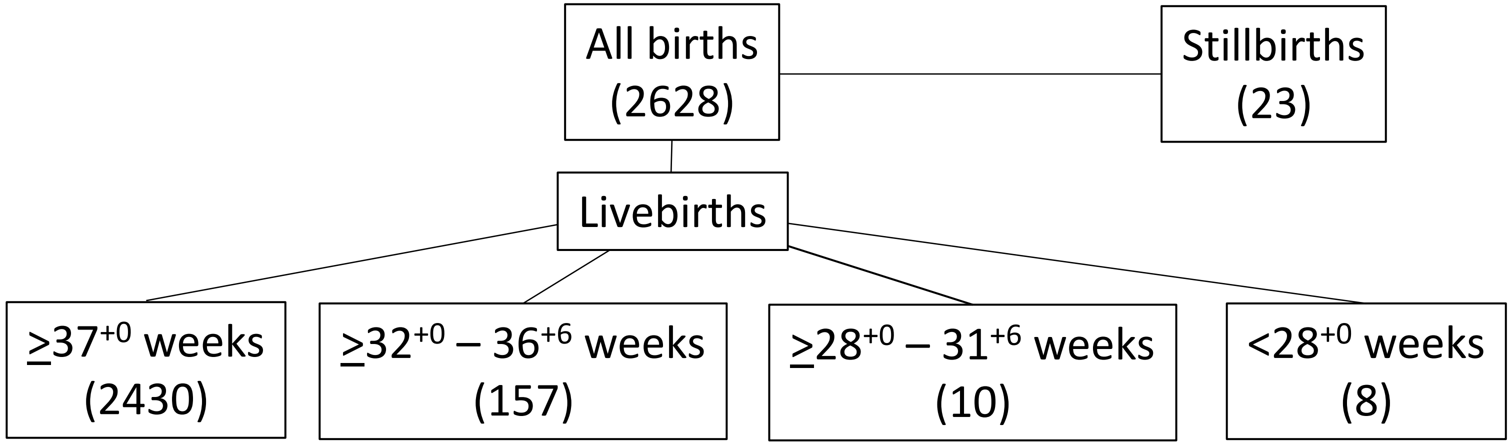

Data of Calgary during pandemic epoch [March 1 to April 30, 2020]

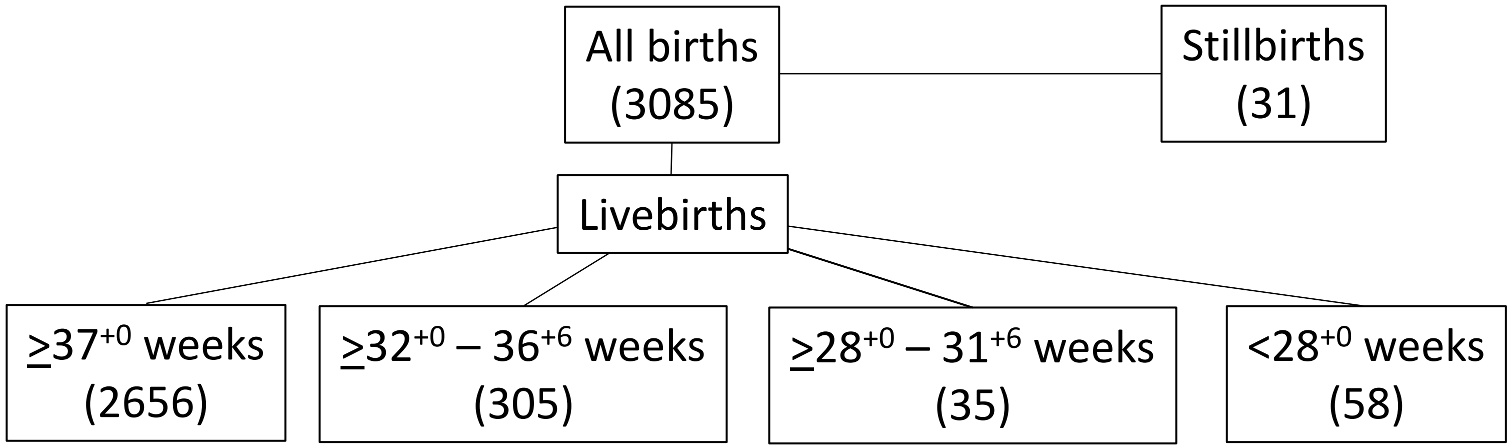

Data of Edmonton during pre-pandemic epoch [March 1 to April 30, 2019]

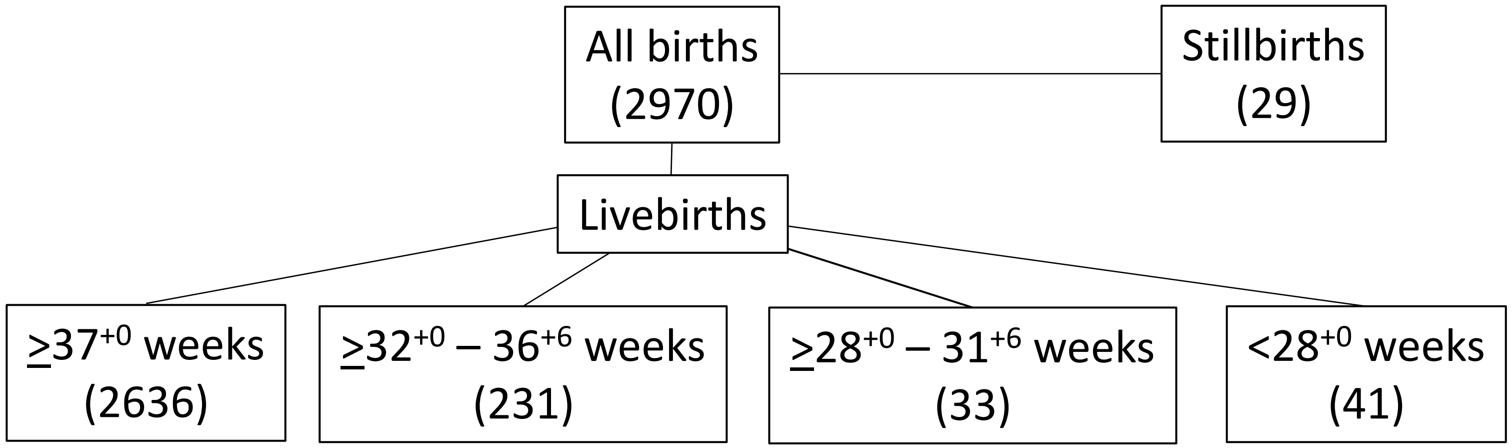

Data of Edmonton during pandemic epoch [March 1 to April 30, 2020]

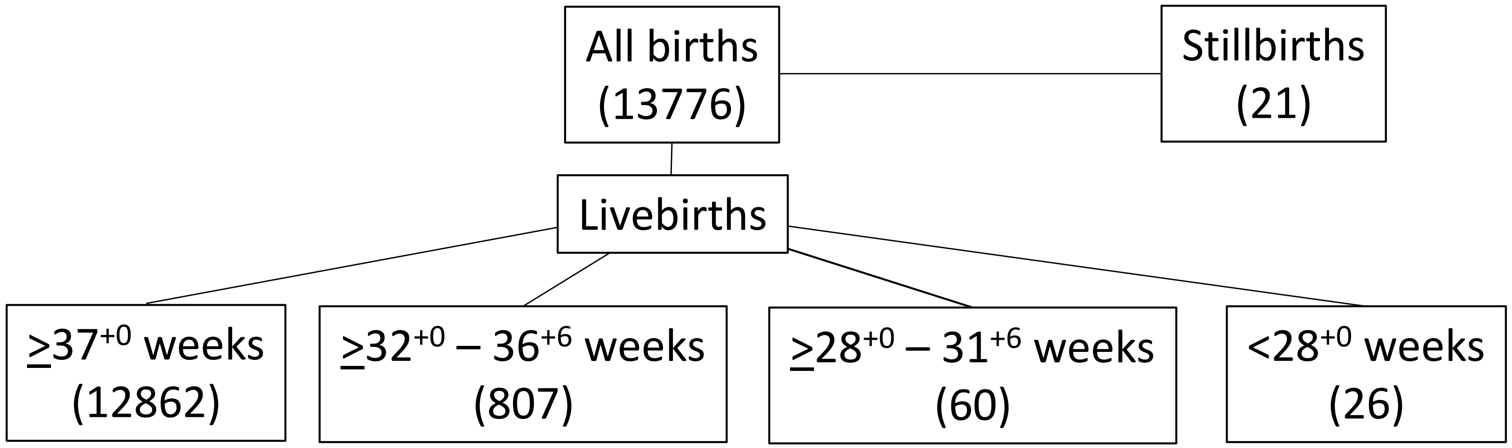

Data of Shenzhen during pre-pandemic epoch [February 1 to February 29, 2019]

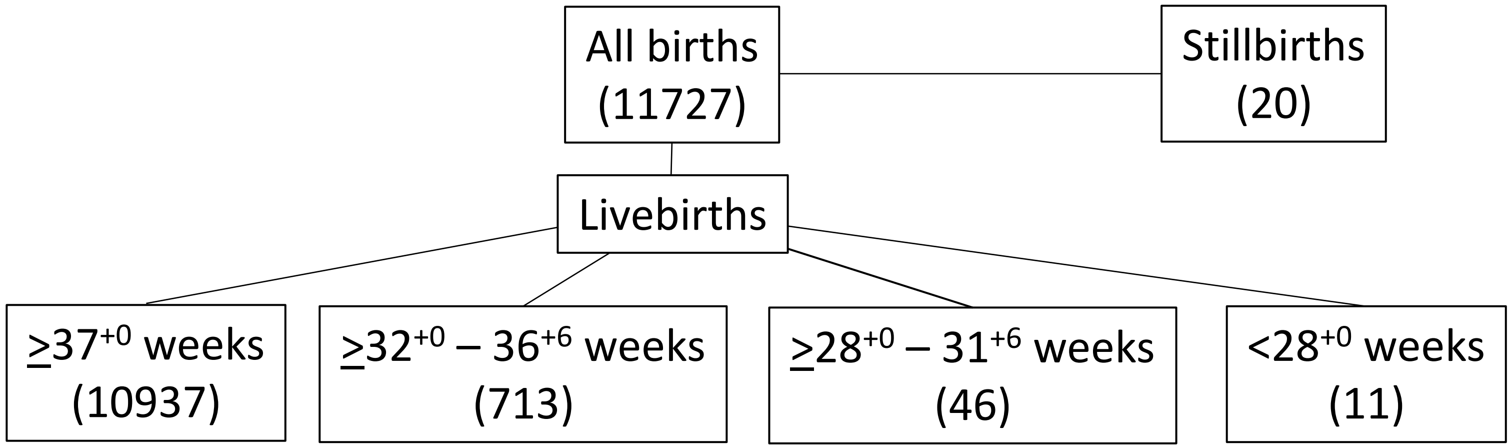

Data of Shenzhen during pandemic epoch [February 1 to February 29, 2020]
